# Patient and public involvement in designing and conducting doctoral research: the whys and the hows

**DOI:** 10.1186/s40900-019-0155-1

**Published:** 2019-08-16

**Authors:** Justine Tomlinson, Kristina Medlinskiene, V-Lin Cheong, Sarah Khan, Beth Fylan

**Affiliations:** 1 Medicine Optimisation Research Group, School of Pharmacy and Medical Sciences, University of Bradford, Bradford, UK; 2 Medicine Management and Pharmacy Services, Leeds Teaching Hospitals NHS Trust, Leeds, UK; 3 Pharmacy, Sheffield Teaching Hospitals NHS Foundation Trust, Sheffield, UK; 4 Bradford Institute for Health Research, Bradford Teaching Hospitals NHS Foundation Trust, Bradford, UK

**Keywords:** Patient and public involvement, Research participation, Online panel, Doctoral studies, Research methods, Collaboration, Engagement, Impact

## Abstract

**Plain English summary:**

Evidence shows that public and patient involvement in research has a positive effect on its quality and end-results. Thus, public and patient involvement in all stages of research is becoming commonplace. There are limited detailed examples however, that describe how to make this possible, especially for those doing PhD research. Doctoral researchers are often new to research practice or have limited experience and are often bound by strict time and financial constraints. It is also not usually a requirement of the award to involve public and patients in their research. Hence, they may not feel confident or motivated to involve or engage with public and patients during their research. We, four doctoral researchers, share examples from our own research studies that have included different approaches to public and patient involvement. Two studies formed public and patient advisory groups who helped design the research questions, data collection tools and recruitment methods. One enlisted the help of an online public and patient panel from a local hospital. A different study worked with patients from an established group to help define key medical words. We did face some challenges, such as the need to develop good group work skills and to apply for grants to cover reimbursement, but we all found it beneficial to involve patients in our studies. We noticed a positive effect on each study’s progression and an improvement in our own self-esteem. In addition, having public and patient involvement helped reduce the isolation we felt as doctoral researchers. Thus, we strongly encourage more doctoral researchers to involve public and patients in their studies.

**Abstract:**

Public and patient involvement (PPI) has been shown to have a positive impact on health and social care research. However, adequate examples describing how to operationalise effective PPI, especially in doctoral studies, are lacking. Hence, doctoral researchers new to research, or those with limited experience, can be discouraged from facilitating PPI in their research. This paper aims to describe and discuss in detail the approaches used by four doctoral researchers to incorporate PPI at different stages of their research studies from study design to disseminating findings.

We aim to inform other doctoral researchers about the challenges and limitations relating to PPI that we faced. Through these, we share pragmatic recommendations for facilitating PPI during doctoral studies.

The description of four case studies demonstrated that PPI could be incorporated at various stages during doctoral research. This has had a beneficial impact on our research study progression, researcher self-esteem and lastly, helped alleviate researcher isolation during doctoral studies.

## Background

There is a growing consensus that public and patient involvement (PPI) in research is instrumental in improving the quality of research projects and strengthening their relevance and impact. These positive effects are reported during all stages of a study from design to dissemination [1, 2].

PPI ensures a focus on topics that concern health service users the most [3–5], and conversations between PPI and researchers promote learning for both parties [6]. PPI has also helped create participant documentation and adapt academic language into plain English, thereby widening accessibility. Presentation of findings by PPI groups can also encourage further implementation of research findings and more effective dissemination [7, 8]. These positive developments have contributed to a significant drive to embed PPI within health and social care research, and it is becoming a prerequisite in funding applications [9].

In addition to these benefits, PPI members have reported that they felt empowered to contribute to society, gain new skills, and were provided with a mechanism to share their experiences whilst directly influencing change [6, 9, 10]. At the same time, researchers reported gaining a greater understanding of patients’ experiences and an appreciation of the problems they face, hence reinforcing motivation to continue their research [9, 11].

We argue that engagement with PPI activities should be introduced during doctoral study, especially within health and social care research PhDs, as the benefits of PPI are well reported. However, doctoral researchers often have limited skills, knowledge, and experience of research methods and work on their own with strict time and financial boundaries. Therefore, they can be discouraged from engaging with PPI from the start of their research. A lack of published examples of how to operationalize effective PPI [9, 12] may also hinder their confidence and knowledge.

This paper discusses the approaches and purpose of PPI activities of four doctoral researchers (see Table 1) and aims to describe how PPI has been incorporated at different stages of their research. We aim to share experiences and pragmatic recommendations that facilitated the PPI activities through in-depth discussion about what worked and what did not, as well as the benefits and limitations.

**Table 1:** Brief overview of the four doctoral research studies

All four doctoral researchers (KM, JT, VC, SK) are practicing pharmacists within the University of Bradford’s Medicines Optimisation Research Group. Their research interests are focused on improving the safe use of medicines.	
• KM’s study is exploring the barriers and enablers to the uptake of new medicines within health care organisations, using oral anticoagulants for stroke prevention in non-valvular atrial fibrillation as an example. The aim of the project is to produce recommendations for optimising local implementation of nationally recommended medicines. The PPI members involved in the project were patients (middle-aged and older adults) with long-term conditions who were taking the studied medicines.	
• JT’s study aims to design new ways to support post-discharge medicines continuity for older people living with long-term conditions. She has involved four PPI members (three older people with experience of medicines changes at discharge and one informal family carer) throughout her study, who have greatly helped to contextualise the research topic.	
• VC’s doctoral research explored the medicines-related risks of repeated hospital admissions in older patients living with frailty [13], and the management of this with pharmacists’ interventions. Part of her study involved an online survey (Delphi method). VC engaged with an established online PPI panel of 30 former or current patients at her local hospital Trust who regularly reviewed participant research documentation.	
• SK’s study focuses on medicines reconciliation processes within hospital and the patient role. SK worked with an already established PPI panel at her local hospital Trust. Six members of the panel took part in SK’s PPI activities (age range: 44 – 70 years).	

## Defining and refining research questions with PPI

In this section, we will share four examples of how we used PPI to refine (or re-define) research questions to ensure their relevance to end-users of health and social care services and therefore increase the potential impact of the research (see Table 2) [1, 4, 5].

**Table 2:** Summary of approaches used by the doctoral researchers to work with patients and the public

Format	Stage of the project	Type of engagement^a^	Recruitment method	Recruitment timescale	Approach	Benefits	Challenges
Existing patient support group	Defining research question(s)	Consultation	Identified via local patients group search (online) and group leads contacted directly via email	Six weeks from the initial contact to attending the meeting	Focus group, face to face	• Meeting in their own setting; • May not need reimbursement for travel expenses and/or time above what is already paid; • Participants familiar with each other and willing to share their views; • Can be a learning experience for the group; • Possibility to engage with the group for future PPI activities; • Informal.	• Facilitation skills and note- taking required; • Difficulty in finding an existing group aligned with your research; • Lack of flexibility for meeting dates and times.
Forming new PPI advisory group	Defining research question(s)	Consultation	Advertised via email to existing PPI groups or invited participants from the initial PPI consultations	Eight weeks from advertising to attending the first meeting	Focus group, face to face	• Participants may have prior experience of research and undertaken training; • Focus of the group is about the research; • PPI members are part of the research team and thus are invested in the study; • Researcher leads the group; • Flexibility of meetings to match the needs of the research study; • Easier to provide feedback to PPI on their input; • Easier to measure impact of PPI on the study as their input is continuous; • Provides additional and ongoing support for the doctoral researcher alongside supervision team.	• Reimbursement required for travel expenses and time; • Expenses might be required for venue and refreshments; • Time commitment (organising and delivery); • Finding existing PPI members with relevant experiences and knowledge; • Facilitation skills and note-taking required; • Uncertainty of interest at the recruitment stage; • Participants not familiar with each other; • Setting expectations of both PPI and the researcher (we created Terms of Reference documents); • Training and support might be needed for both PPI and the researcher.
	Project development, delivery & data analysis	Collaboration					
Consulting with patients in outpatient clinics	Defining research question(s)	Consultation	Direct healthcare team approached patients after their appointments	Five weeks from initial contact with healthcare team to attending outpatient clinic	One to one interview	• Helps build relationships with the healthcare team within the Trust; • May not need reimbursement for travel expenses and/or time; • No fee for the venue.	• Local Trust’s approval required; • Support from direct healthcare team required; • Participants may be less willing to share their views about the care they have just received; • More difficult to differentiate between PPI and being participants in research for both PPI members and the researcher.
Existing PPI groups at local hospital Trust	Project development	Consultation	Identified individuals from existing PPI panels at local hospital’s Research and Development department via research co-ordinator and advertised via email	Six weeks from contacting PPI panel members to the meeting	Focus group, face to face	• No expenses or meeting venue fee required as already reimbursed by the local Trust; • Panel members experienced in research and are familiar with the PPI role.	• Facilitation skills and note-taking required; • Support required from hospital research co-ordinator; • May only be accessible to hospital staff; • Expenses might be required for refreshments; • Limited time in order to complete activities if one-off meeting.
Existing online PPI panel	Defining research question(s)	Consultation	Identified group at the local hospital's Research and Development department via research co-ordinator and advertised via email	Three weeks from planning to the first PPI activity	Online questionnaire	• Relatively short timeframe from setting up to achieving objectives; • No costs for meeting venue or refreshments; • Panel members already reimbursed by the local hospital Trust; • Potential to access hard to reach individuals e.g. housebound patients; • Anonymous process, which may encourage PPI members to express richer views; • Panel members were able to ‘edit’ participant-facing documents and make suggestions.	• Distance engagement preventing opportunity to correct misunderstandings; • Support required from hospital research co-ordinator; • May only be accessible to hospital staff; • Self-selected members due to digital technology capacity and capability; • Documents must be ‘readable’ online.
	Project development	Consultation					

^a^ As per INVOLVE definitions for type of engagement [14]

PPI during research design can have a positive effect by improving its relevance as well as increasing the researcher’s understanding of the patients’ experiences [3, 9]. It is therefore valuable for PPI members to have experience and knowledge of the research area under investigation in order to achieve a meaningful input. However, researchers should not severely limit their recruitment of PPI members with specific criteria, to ensure they are able to identify potential PPI members [6]. Hence, this can be challenging for doctoral researchers commencing their study, as most will not yet have a clear research question, understand the potential outcomes of PPI, not know how and where to access PPI, and may find it difficult to communicate research terminology to a lay audience [9].

### Working with existing patient groups

One way to facilitate PPI is to approach an existing patient group that relates to your research area. In our first case, KM engaged with an existing local support group for patients with atrial fibrillation. KM identified the group through an internet search of hospital patient support groups, charity groups and general patient groups and then contacted group leads directly via email. She met with the group’s lead member to discuss the potential role of PPI in her research, using information (e.g. the Public Information Pack [15] from INVOLVE in England or Involving People (Welsh equivalent)) to guide the conversation. The group lead then discussed the information with the group members and obtained their consent for the researcher to attend one of their regular meetings. JT, VC and SK approached established PPI groups who already had knowledge about engagement in research.

KM attended one of the group’s regular meetings, at which six members were present. After introductions and a general discussion about PPI, group members were verbally asked open questions allowing them to comment and share their experiences of initiating and taking oral anticoagulants. As the discussion evolved, questions were narrowed down to focus on specific aspects of the study, e.g. methods, recruitment strategy, proposed study plan. The two-hour discussion highlighted the potential benefit of the study to health service users. It also became apparent that additional healthcare professional groups (e.g. nurses, pharmacists) should be included in the data collection stage as they played an important role in these patients’ care. Although specific study outcomes were not identified at this stage, early PPI provided insight into patients’ experiences, which were used as a springboard to further project development.

KM experienced several advantages to engaging with this established patient group. Members of the group had experiences of and perspectives on their condition and treatment, they knew each other, which meant they felt comfortable in discussions and thus readily engaged in sharing their views. Their meetings took place in an informal environment, which helped to put KM at ease. Furthermore, they were happy to discuss the project as part of the group meeting so additional travel costs were not incurred. Nevertheless, conducting an effective patient group discussion was challenging and good facilitation skills were required to keep the conversation focused and on track, which has also been highlighted by other researchers [6]. Printed study materials prepared in advance, outlining key questions, proved to be useful in obtaining answers to specific lines of enquiry.

### Forming a patient advisory group

An existing patient support group relevant to your study may not be available in your area or may not exist. This was the case in JT’s research and thus she decided to form an advisory group specifically for her study. Four older individuals with experience of medication changes at hospital discharge were recruited from existing PPI groups within the University, local hospital Trusts and Clinical Commissioning Groups. These groups were specifically targeted as the members would have already been involved with research activities and would therefore have an understanding of research processes. A flyer was developed to facilitate recruitment of PPI group members. This briefly explained the project, what the group would be asked to do and the types of experiences she was interested in finding out about. Individuals were asked to respond by email or telephone call. JT contacted those who expressed an interest to explain more about the study and the aim of the group, the level of commitment (including expectations of the role), and reimbursement. This helped her to build rapport with group members right from the outset.

Two interactive workshops with the four members were organised over a period of two months and were facilitated by JT. These three-hour sessions focused on exploring the group’s experiences of medicines-related care after discharge and developing a timeline of post-discharge medicines events that led to development of the research question and study aims. The emotions that the group expressed about the research topic were exceptionally powerful and motivated JT to redefine her topic from the patient perspective. The sharing of these experiences helped the group to bond as they found commonalities and were able to offer support to each other. The group also voiced that the topic resonated strongly with them and they were all driven to be involved to make a difference to current healthcare practices. This demonstrated to JT that the topic was worth investing in. The findings from these workshops added strength and direction to the study and informed a subsequent successful grant application (which included funding for continued work with this advisory group). The main challenge of creating a PPI group specifically for the study was the cost of reimbursing members, refreshments, room booking and travel expenses (in line with INVOLVE guidance [14]). This was overcome by obtaining a small grant from the local NIHR Research Design Service to support PPI work during study design. Another challenge, as highlighted by KM, was the need for strong facilitation skills to guide the discussions. Ensuring a clear focus and aim for the workshop at the outset and defining expectations (of both the PPI members and the researcher) helped in this situation. In response to this, JT created role description documents.

### Consulting with patients in outpatient clinics

The examples discussed above involved focus group-type discussions with PPI members. An alternative approach is one-to-one discussions, which was initially piloted by KM. After obtaining approval and support from an outpatient arrhythmia clinic and the hospital Trust’s Research and Development team, patients were invited to speak with KM after their outpatient appointment. Although patients were happy to engage in short discussions about the study, KM felt that they were more reserved in sharing their views than participants in the existing patient group. Nevertheless, it was still possible to assess which factors were important to patients in order to shape the research to be more patient-centred. However, on reflection, KM believes PPI activities through a group discussion worked better as it provided greater insight into patient experience and views.

### Using an online patient panel

In light of increasing digital technology use, online PPI panels are becoming widely available to researchers and offer an alternative approach of conducting PPI activities [16]. There is widespread evidence of the exclusion of older people as well as under-recruitment of older people into research [17]. There is also a dearth of evidence which examines PPI activities in older people living with frailty. Previous research has shown the value of planning and logistics in effective recruitment of older people to PPI and research activities: for example, carrying out PPI activities at the participants’ usual place of residence can facilitate involvement by reducing burden [17, 18]. VC explored accessing an existing frailty panel (the patient group most aligned to VC’s research) within her local hospital Trust but at the time, this panel was not yet established. The frailty panel is expected to consist of members who are community-dwelling with limited mobility, where PPI activities would take place in the patient’s home. The hospital Trust will provide support and guidance to researchers wishing to access the frailty panel by accompanying the researcher into the patient’s home to carry out PPI activities. The unavailability of the frailty panel at the time prompted VC to identify alternatives. The hospital had an existing online public advisory panel of 30 former or current patients, which VC thought was suitable to access due to the eventual online nature of her project (online Delphi study).

The consultation with the online panel was carried out over a three-week period, whereby VC prepared documents including questions to the panel, and they replied electronically with their response. The research co-ordinator at the hospital Trust where VC had an honorary contract collated the responses and returned them to her. The first phase of the online PPI process involved the preparation of a plain English summary of the research topic and the panel was asked to provide feedback relating to two main areas: importance of research area to patients and acceptability of proposed research methods.

Six panel members were involved in this exercise. All panel members thought the research was clinically relevant and beneficial for patients but expressed some views about the need for clear dissemination of study results. The panel raised a number of concerns and suggestions regarding the proposed research method. For example, they suggested increasing the sample size after highlighting that attrition would be high in the target patient population. The panel also thought that carers of patients should be included in the study. Overall, the online PPI panel provided useful feedback, particularly to this study, which used online documents to collect research data. It also helped to highlight some of the potential difficulties in the recruitment and retention of participants, therefore encouraging VC to consider strategies to help minimise this.

The online consultation process also provided anonymity, allowing the panel members to fully express their views, which some may prefer. No reimbursement for hospitality or transport was required for panel members in this instance. Additionally, the panel members were already being reimbursed by the local hospital Trust, thus additional payment for their time by the doctoral researcher was not required. This can be beneficial for financially constrained doctoral projects. The downside to this approach was that wider exploration of PPI views could not be conducted at the time and VC needed to carefully establish and articulate what it was she required from the panel at the outset. Access to this online PPI panel was also limited to hospital staff members.

## Project development

Designing and reviewing participant information sheets (PIS) and consent forms are common PPI activities during project development [3, 19]. We have used two approaches to involving PPI in study development which are presented in the section below: consultation with the online panel and co-design with PPI advisory groups (see Table 2).

### Development of study documentation

#### Advisory group

Having found PPI beneficial during the design of her study, KM formed an advisory group of three members; two members from the initial support group consultation and the third member from a PPI member panel of another large research programme within her research group. The PPI members were provided with a Terms of Reference document stating objectives and expected commitment. They were reimbursed for their travel and time in line with INVOLVE guidance [14]. Providing this information at the start proved to be useful in managing expectations of both the group and the researcher.

In the first meeting, the PPI advisory group was involved in co-designing a PIS and a participant consent form. In a two-hour meeting held at the University, the group was presented with information that should be included within a PIS by showing them a conventional template (presented as A4 pages). The first task involved a review and gathering of opinion about the wording and terminology. Several compromises were made as some information (thought to be crucial by the researcher) was recommended to be omitted by advisory group members. For example, statements regarding storage of interview recordings and transcripts. The completion of the first task resulted in a significant reduction in word count and improvement in wording of the text.

The next step was to consider how the information could be presented in a more participant-friendly format. KM encouraged group members to voice their ideas so that a novel design could emerge. The group felt that reading two or three pages of A4 text was 'boring', and they suggested an alternative format of a brochure. Other changes included: use of colour and images, including a picture of the researcher to make the PIS more welcoming, and including flow diagrams explaining what participation would entail. After this meeting, a new version of the PIS was drafted. It was posted (or emailed) to the advisory group for further review and comments. After a second round of re-design, the final co-produced PIS was agreed. Designing the PIS with the advisory group led the development from a conventional three-page A4 document to a trifold brochure full of colour, easy to read text and flow diagrams. Additionally, this process of co-design with the group added to developing KM’s skills as a researcher, e.g. developing patient-friendly study information, and co-ordinating and facilitating group work. Using this approach was also a very clear way to demonstrate and feedback to group members on their valuable and impactful involvement, thus building their confidence for further involvement in other aspects of the project.

#### Online panel

The online PPI panel was contacted, and seven members, including two from the initial round of consultation activities, were engaged to assist with aspects of project development and to advise on the readability of participant-facing materials (e.g. consent form and PIS) for VC’s study. After two weeks of the initial PIS being available online, responses were obtained from all panel members, who offered comments which led to changes to the research process, the timescale for the start of the study and the language used within the documents. This activity resulted in the re-design of the PIS so that there was more emphasis on the benefits for participants and more direct language was used such as referring to the participants as ‘you’ instead of ‘they’.

### Defining terminology

Translating specific terminology into plain English can be challenging. Patients can be confused and may not understand medical terminology or jargon such as ‘medicines reconciliation’, which may act as a barrier to taking part in the research [20, 21]. This is also an important factor when considering informed consent. Participants must fully understand the study details in order to decide whether to take part. One way to overcome this is to use PPI to re-define terminology used within health and social care research [21].

To ensure her research focusing on medicines reconciliation resonated with and was fully understood by participants, SK used PPI input to design and develop a definition of medicines reconciliation to be used in patient information documentation and during patient interviews. This consultation exercise aimed to work with patient and public representatives to establish their current understanding of the term “medicines reconciliation” and agree a user-friendly definition.

SK was able to form a consultation panel from an existing PPI group at her local hospital Trust where she had an honorary contract. She initially created a short document detailing her study, why PPI input was required and an invitation to a one-off group meeting. Facilitated by the Trust’s research co-ordinator, all members of existing PPI panels linked with the Trust were sent the document via email and were asked to contact SK if they were interested in attending. PPI members needed to have experience of a hospital admission to take part. SK arranged a date for the meeting that suited all six interested members. This process from initial email to meeting date took six weeks. No additional reimbursement by SK was required for attending the group as the members were already being reimbursed by the hospital Trust. However, lunch was provided as a way of thanking the members for their input. SK used Nominal Group Technique methods [22] for her meeting, incorporating engaging and interactive activities to spark interest.

The meeting was carried out in five stages [23] over two and half hours:

- *Stage 1- Idea generation (PPI members spent time generating ideas relating to the question posed)*
- *Stage 2- Round Robin (PPI members took turns to read out their ideas and all were collated)*
- *Stage 3- Clarification (The group discussed the idea more widely and duplicate ideas were removed)*
- *Stage 4- Voting (Ideas were ranked in order of priority by the PPI members)*
- *Stage 5 – Action (The group decided the best way forward based on the results of the vote)*

Following this approach [23], each PPI member was able to develop their own unique definition of medicines reconciliation. The final definition that was created by the group was agreed to be easy to understand and to effectively define medicines reconciliation, however it was felt by the group to be too long to be used during interviews. Following review, the attendees agreed on a shorter definition.

SK found this activity an effective and valuable way to break down an everyday medical term and replace it with an easy-to-understand definition. SK has since used this definition in her study. Five of the six members reported finding the session useful, one was unsure. Feedback from the PPI members included increasing the length of the meeting and giving more information beforehand to allow attendees to prepare. SK also found it challenging to set the meeting date to suit members’ availability, which accounted for the six-week set-up time.

### Developing a data collection tool

In addition to developing patient-facing documentation, PPI can also be successfully engaged in the design of data collection tools. To maximise the uptake of her data collection method of diaries, JT involved her advisory group in a co-design process that allowed them to generate ideas and create something that could be used by her study population. Involving her advisory group with this task was incredibly beneficial as they were able to consider the level of participant burden of using a diary and therefore the impact on the research. From the outset, the group advocated that carers should also be allowed to fill in the diary and they developed plain English instructions for participants to ensure that the procedure for completing it was clear. They also created example entries to help participants know how they should complete their diary and decided to call it a “My Medicines Journey Notebook”, as this was felt to have better connotations than ‘diary’ or ‘journal’. During initial stages of design, the group critiqued examples of other diary tools that had been used within research and were able to use these to make suggestions for content. From this exercise it was decided that a one-page, semi-structured entry format with specific questions would be the best compromise for participant burden and research quality. JT then developed a draft of the notebook based on these ideas and there were four iterative rounds of feedback and re-design with the advisory group. Once finalised, a member took the notebook to their luncheon group and asked their peers (average age 83 years) to review the design, layout and language used to ensure that it was appropriate. These peer discussions led to changes in the size of the document from A5 to A4 with a minimum font size 14 as some struggled to read anything smaller (whereas the group had originally requested A5 and size 12 font). Overall, this co-design activity has been thought to support participant engagement with the method and has encouraged use of the diary.

## Delivery

Here, we discuss two approaches of involving PPI in the delivery of our studies that have been used to date: participant recruitment and validating literature review findings [3, 7, 24].

### Participant recruitment

PPI advisory group members were an integral part of KM’s patient recruitment strategy. The group was familiar with the study through the development of the PIS and consent form and was therefore deemed well-equipped to identify eligible patients. They distributed study information and approached eligible participants from within their patient support network. Interested participants were asked to contact KM for more information. PPI members were able to recruit harder to reach individuals (e.g. age and taking a certain medicine) into the study. It is important to note, identification and recruitment of study participants by PPI members, just like other recruitment approaches, requires ethical approval.

Additionally, KM found that developing her recruitment strategy with the PPI advisory group was useful as they had wide links with local community groups, e.g. church groups and charity groups, which provided additional resources for consideration. Furthermore, PPI who are active users of the studied service will be consulted to help identify appropriate healthcare professionals who can be approached by KM for recruitment in later stages of the study. Overall, using PPI in developing recruitment strategies and recruitment of study participants is well worth considering, especially when recruitment is expected to be challenging.

### Reviewing credibility of literature review synthesis

KM has involved the PPI group to review her literature review narrative synthesis results to ensure its credibility. The group was presented with themes and sub-themes of the narrative synthesis, which were briefly explored and explained. The discussion refined the themes (i.e. wording) and confirmed the findings to be reflective of the PPI members’ experience. This exercise also identified additional aspects that were not present in the literature review to explore in the interviews with participants.

## Data analysis

PPI has also successfully been integrated into data analysis [22]. A study by Ellins et al. identified that when older people are involved in data analysis, “...they make sense of the findings using their own perspectives” ([24], p.32). Others have also highlighted the beneficial impact of involving PPI members with data analysis [25]. JT has involved her advisory group during the analysis of patient and carer’s interview transcripts. The group were able to add their alternative perspectives and provided insightful interpretations of the data. JT hopes that this will result in a better representation of the findings that will be more relevant and accurate. When asked how they felt about this task, the members were keen to be involved, however, did not feel that they wanted to read through full interview transcripts as this felt an overwhelming task. Instead they requested to review and discuss a variety of anonymised excerpts, chosen by JT. They also suggested a need for some basic training in data analysis to build their confidence and improve academic rigour.

KM provided her PPI advisory group with two printed anonymised interview transcripts at one of the regular meetings. The transcripts were intentionally selected to represent interviewed participants with different experiences to stimulate discussion. Firstly, KM explained to the group the data collection process using interviews and thematic data analysis principles. Then, the PPI group members commented on transcripts while reading them using a ‘think aloud’ approach. The researcher facilitated the discussion by capturing their ideas on the flipchart and probing for further discussion. The workshop took three hours and the output was a mind map encapsulating PPI members’ thoughts on the transcripts. The mind map was then used to develop a thematic framework for the analysis of interview transcripts by the researcher. The last stage of the analysis will involve PPI advisory group members discussing themes and sub-themes developed by KM after completing the data analysis.

## Dissemination

There is evidence to support involving PPI in dissemination of research findings, as they are able to provide access to a wider audience than traditional academic circles. They are also able to translate findings into meaningful, everyday messages and demonstrate relevance to patients [7].

Within her study, JT will involve her advisory group members in local dissemination events that will include stakeholders, healthcare professionals, third sector organisations and members of the public. During these events, the PPI group will share their interpretation of the research findings and what they could mean for future patients and their carers. JT also aims to co-present the findings with the PPI members at academic conferences.

PPI can also help develop accessible summaries of research findings, which can be disseminated to research participants and the wider public [7, 9]. Other studies have shown that PPI may develop more creative methods of dissemination than the researchers [25] or may have access to groups and forums that the researcher is not aware of [26]. KM’s study participants expressed interest in receiving a summary of the study findings and thus the PPI advisory group will help with writing a summary of the findings from the interviews with patients in concise and plain English. The co-design approach used with the PPI group during the earlier PIS design will be employed.

Other reported dissemination approaches with PPI members include involvement in report writing, co-authoring academic and newsletter articles, giving seminars, or through social media, e.g. blogs [14, 27].

## Discussion

We have demonstrated that doctoral researchers can incorporate PPI within their studies and have provided detailed examples of how to create or access PPI groups and the types of activities that can be conducted. Pragmatic suggestions of how PPI can be engaged at all stages of a study are provided in Table 3. Our studies involved PPI members who were middle-aged or older adults with long-term conditions. Therefore, the discussed approaches might not be suitable for working with children, young adults, or adults without long-term conditions.

**Table 3:** The 10 top tips for working with patients and the public in doctoral studies

1. Use the internet and link with other institutional resources to identify your PPI members e.g. search for existing PPI panels, support groups, expert patients, established hospital groups, online panels, and University panels.	
2. Before commencing any PPI activity, have a clear understanding of your aim for PPI. It is useful to create Terms of Reference for groups you establish and refer back to them regularly.	
3. Follow INVOLVE guidelines for reimbursement and travel expenses and consider how payments may impact on your members’ benefits and tax, if applicable. If you do not have funding available, search for grant opportunities to enable you to conduct PPI work (e.g. Research & Design Service in UK).	
4. Offer refreshments or catering at your meetings. We have found that an informal social lunch helps members bond and feel valued.	
5. Carefully plan your sessions to maximise your time together. Sending agendas and background information before your session will help ensure everyone remains focused on the topic.	
6. Consider carrying out a training needs analysis with your PPI group to identify if they require any training before undertaking research tasks.	
7. Ask your members how they prefer to receive information from you and in what format; email or post, size of text, colour and so on.	
8. Provide regular feedback on how you have used PPI comments so people are able to recognise their contribution to your work. To achieve this, make regular notes during and after the meeting with PPI to capture their input.	
9. Do not underestimate the time you will need to plan and carry out PPI activities.	
10. Be creative during your meetings. Suggest documenting ideas on sticky notes or flip chart paper and use a variety of activities to help all group members to contribute.	

Our experiences support the reported benefits in the wider literature [1–9, 12]. In addition, we have found that PPI contributes to the personal development of doctoral researchers on their journey towards becoming independent researchers. Incorporation of PPI in doctoral studies, especially during the earlier developmental stages, enabled us to get out “into the field” and engage with people about real-life experiences. Our PPI advisors have become ‘critical friends’ and through developing these rich relationships we can see the relevance of our work. This has had a beneficial impact on our self-esteem and provided us with an additional support mechanism during our doctoral studies, which contributes to improving our well-being [28].

PPI however, is traditionally not seen as “original empirical research” and thus does not fit the assessment criteria of doctoral studies. Due to strict time and finance rules, doctoral researchers may not see the need for PPI in order to gain their qualification or may not prioritise this type of work, instead favouring to move straight into their ‘formal’ research. We were aware of the potential benefits for PPI engagement and were encouraged to conduct PPI activities by our supervisors. We did however encounter time and financial pressures, which are well documented challenges [2, 3, 19]. Therefore, in order for us to carry out our PPI activities we needed to apply for additional funding to reimburse PPI members, purchase refreshments, book rooms or partner with local organisations to reduce the financial burden. This required planning and time, and without adequate support could result in undue stress, decreasing the well-being of doctoral researchers [28].

Other reported barriers to conducting PPI activities include poor recruitment, low meeting attendance, and inadequate management of the researchers’ and PPI members’ expectations [2, 3, 19]. Although in our examples we achieved target recruitment and good meeting attendance by using strategies listed in Table 2, we needed to have a clear understanding of our aim for our PPI. Thus, creating Terms of Reference for groups and referring to them regularly was a useful strategy. Additionally, it is often difficult to involve a diverse range of people in PPI activities and research teams have often relied on a few selected individuals, reducing the representativeness of the input [2]. For instance, older people and those from traditionally marginalised groups, e.g. people living with dementia or non-English speakers have historically been excluded from being involved in research design. Furthermore, appropriate training, support, and funding is required within doctoral studies to encourage and facilitate PPI activities, preventing them from becoming a tokenistic or ‘box-ticking exercise’ for meeting the requirements of funders or ethics panels [19].

Another important aspect of PPI activities in research is evaluation [12]. There are two main themes of PPI evaluation: impact on the project and experiences of the PPI journey, including researcher-PPI relationship. Staley and Barron [6] debate that what the researcher learns from their conversations with PPI is subjective and unpredictable, hence evaluation can be tricky. They suggest evaluation could begin with a key question: ‘Does the interaction between researchers and the public lead to change?’ [6]. Whilst we evaluated the impact that PPI involvement has had on our studies and what changes we have made as a consequence (see Table 4), and collected feedback to improve the ongoing process of PPI activities, we found formal evaluation of PPI experiences challenging (especially in the scenario of smaller groups). We each completed brief evaluation of activities (e.g. anonymous feedback forms): SK and VC were able to collate feedback on how they ran their activities and anonymous surveys worked well with their PPI members as these were one-off group events. Also, due to the nature of the activity they did not build long-standing relationships with their PPI, perhaps making it easier for the members to be critical of their experiences. This is in contrast to the small advisory groups set up by KM and JT who met regularly and had developed rapport over time. The anonymity of respondents could not be ensured, and we question whether our members felt that they could offer unbiased feedback. Although evaluation of PPI activities is important, measures of success at doctoral level need further consideration. In this article, we have chosen to give our personal reflections of the insights we have gleaned and demonstrated what we have changed, by way of evaluation, which we feel adds value to the field.

**Table 4:** Examples of direct changes made to our studies during project development consultations with PPI members

Comments from PPI members	Direct changes made by researcher
You may experience recruitment challenges as you are involving busy wards and clinical staff	Questionnaires were made as simple as possible, with realistic timeframes given to complete, accounting for holiday periods
You don’t define ‘interventions’ so it is difficult to understand what you are going to ask	Additional wording was developed to clarify the term ‘intervention’ and added to the PIS
The Delphi process is not clear to me: patients are asked to revise their answers at every round? How is the agreement reached: are they forced to choose among the most chosen answer, even if they were not their first choice?	Additional wording added on PIS to clarify: “You will get a summary of the survey results so that you have an opportunity to review your previous score and if appropriate, change your score or add further comments”
The PIS is too lengthy and older patients may not be able to read it all	PPI members suggested using a Part1/ Part2 style of PIS, whereby Part 1 gives a brief overview, allowing patients to decide whether to continue reading on if they are interested
The PIS is too wordy and may deter people from taking part	Changed format to a patient-friendly, colourful brochure and prioritised wording to illustrate the points that PPI highlighted as important to them
Some older patients may prefer pictures rather than text to help them understand the research	The group designed a pictorial version of the research journey which was included within the PIS and used to illustrate the study when discussing with potential participants
At what time points will you interview patients?	PPI members mapped out their experiences of post-discharge events (e.g. when medicines are usually delivered, when GP reviews are held). Interview milestones were then agreed at 2 weeks, 2 months and 6 months post-discharge. Participant burden was also discussed and it was advisable to let the patient settle in at home for at least a week before contacting them
Interviews are estimated to take one hour long, this is felt to be appropriate in our experience	Interviews were described as no longer than 60 min in length in the PIS
Our experience is that patients also see nurses and pharmacists alongside doctors in the clinic	Recruitment strategy was adapted to include nurses and pharmacists
Reimbursement for participation in the research needs to be accessible for patients	The PPI members agreed which high street gift voucher options would be of most value to participants

## Conclusions

Overall, PPI has a potential to positively contribute to the development of both doctoral studies and the doctoral researcher. Alongside these benefits, our PPI members voiced that taking part in our activities fulfilled their wishes to help other patients and topics were interesting to them. Furthermore, JT’s advisory group all agreed that they are “big supporters of health research where they feel they can make a difference and contribute their voices to important topics”.

We believe there should be greater uptake of PPI within doctoral studies with adequate support (e.g. time, training, funding) by making PPI integral to the design of doctoral research proposals. We hope our experiences will encourage doctoral or even well-seasoned researchers to consider incorporating PPI within their work in a meaningful way and build a PPI ethos within their research groups. Further work needs to focus on better methods for gathering PPI perspectives of their experiences and how we successfully evaluate during doctoral research.

## Data Availability

Not applicable.

## Abbreviations

NIHR: National Institute for Health Research
PIS: Participant information sheet
PPI: Public and patient involvement
